# Target and peripheral dose from radiation sector motions accompanying couch repositioning of patient coordinates with the Gamma Knife^®^ Perfexion^™^

**DOI:** 10.2478/v10019-011-0012-9

**Published:** 2011-04-23

**Authors:** Tuan-Anh Tran, Vincent Wu, Harish Malhotra, James P. Steinman, Dheerendra Prasad, Matthew B. Podgorsak

**Affiliations:** 1 Department of Radiation Medicine, Roswell Park Cancer Institute, Buffalo, USA; 2 Department of Molecular and Cellular Biophysics and Biochemistry, Roswell Park Cancer Institute, Buffalo, USA; 3 Department of Physiology and Biophysics, State University of New York, Buffalo, USA; 4 Department of Neurosurgery, Roswell Park Cancer Institute, Buffalo, USA

**Keywords:** gamma knife, perfexion, shutter effect, stereotactic radiosurgery, dosimetry

## Abstract

**Background:**

The GammaPlan^™^ treatment planning system (TPS) does not fully account for shutter dose when multiple shots are required to deliver a patient’s treatment. The unaccounted exposures to the target site and its periphery are measured in this study. The collected data are compared to a similar effect from the Gamma Knife^®^ model 4C.

**Materials and methods.:**

A stereotactic head frame was attached to a Leksell^®^ 16 cm diameter spherical phantom; using a fiducial-box, CT images of the phantom were acquired and registered in the TPS. Measurements give the relationship of measured dose to the number of repositions with the patient positioning system (PPS) and to the collimator size. An absorbed dose of 10 Gy to the 50% isodose line was prescribed to the target site and all measurements were acquired with an ionization chamber.

**Results:**

Measured dose increases with frequency of repositioning and with collimator size. As the radiation sectors transition between the beam on and beam off states, the target receives more shutter dose than the periphery. Shutter doses of 3.53±0.04 and 1.59±0.04 cGy/reposition to the target site are observed for the 16 and 8 mm collimators, respectively. The target periphery receives additional dose that varies depending on its position relative to the target.

**Conclusions:**

The radiation sector motions for the Gamma Knife^®^ Perfexion^™^ result in an additional dose due to the shutter effect. The magnitude of this exposure is comparable to that measured for the model 4C.

## Introduction

The Gamma Knife^®^ Perfexion^™^ (Elekta Instrument AB, Stockholm, Sweden) has 192 ^60^Co sources mounted onto eight sectors, forming a partial conical shape inside the Gamma Knife unit.^1^–^5^ The unit includes a Patient Positioning System (PPS) that automatically positions the patient’s head to the coordinates of a treatment run by moving the entire couch apparatus to which the patient’s head is attached. Before any patient motion (either to the initial treatment position, between consecutive shots, or to the setup position after the final shot), the eight sectors within the unit move to the shielded “sector off” position. When treatment begins, the PPS moves the framed head to the planned treatment coordinates, the radiation sectors move into the appropriate collimator position, and the prescribed dose is delivered.^1^–^5^ The purpose of this study is to evaluate the extent of exposure to patients that is associated with these motions; in particular, with the motions associated with the transition of radiation sectors between the “sector off” position and the open collimator position. The importance of correct treatment dose in radiation therapy^6^, especially hypofractionated treatments^7^, has prompted many studies into the accuracy of dose delivery with Gamma Knife radiosurgery.^8^–^11^

There are three sources of undocumented dose during a typical treatment with the Perfexion^™^: the transportation dose from leakage and scatter, the leakage and scatter dose during patient positioning between coordinates, and the shutter dose. The transportation dose results from the exposure the patient receives while moving between the setup and treatment position at the beginning and end of a run. Even though the sources are shielded in the “sector off” position during this phase, the shielding doors are open and the patient is exposed to leakage and scatter radiation. While the sectors are in the “off” position, when the PPS undergoes change in treatment coordinates, exposure will result from leakage and scatter from the sources. Finally, the shutter dose results from exposure when the radiation sectors move between the collimator position and the “sector off” position; this occurs before and after the PPS changes shot coordinates as well as before and after a treatment run.

There is considerable emphasis on conformal dose planning, with associated conformity indices providing a quantitative dosimetric quality measure for a radiosurgery treatment plan. Because of the irregular shape of many targets, treatment plans usually call for a considerable number of isocenters to deliver a conformal treatment, resulting in the use of multiple shots requiring multiple repositioning by the PPS with multiple sector transitions. The consequence is the potential for considerable, undocumented dose, the degree of which is evaluated in this work. With the introduction of the Perfexion^™^, there have been several studies comparing it to its predecessors.^1^–^3^,^12^–^14^ In this study, we compare our results to a similar study analyzing added dose from repositioning with the Automatic Positioning System of the model 4C.^15^

## Materials and methods

### Setup for measurements with an ionization chamber

**FIGURE 1.**
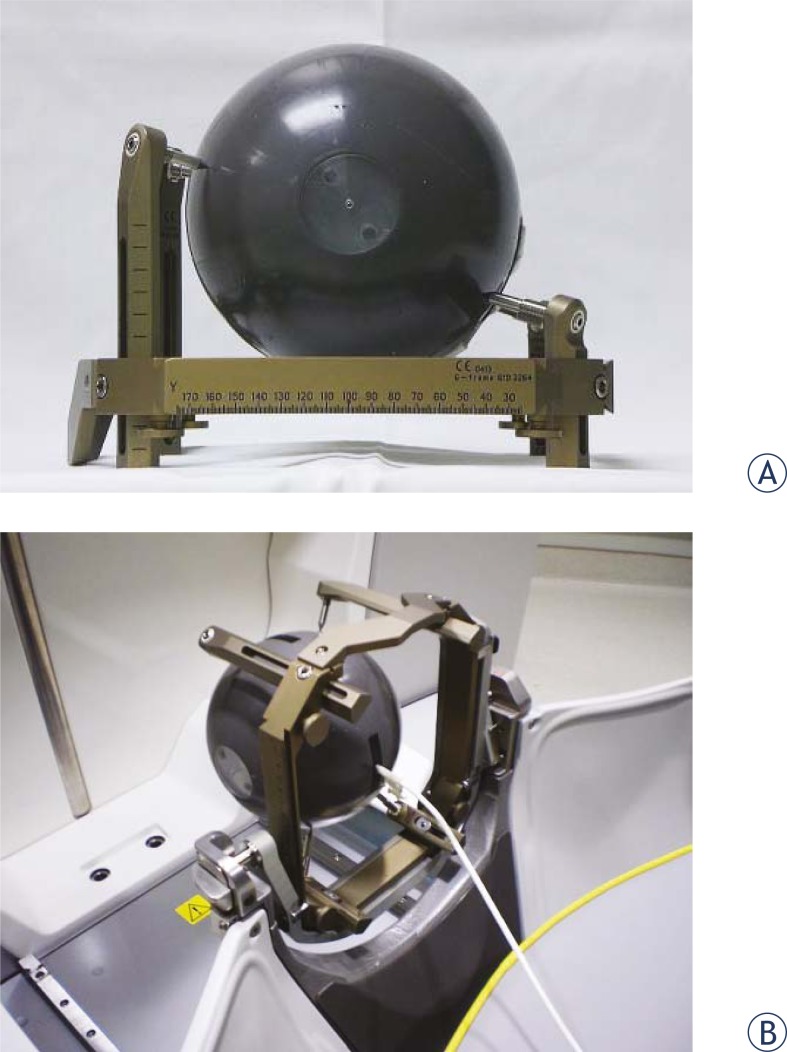
Setup of phantom for measurements. A: Framed phantom B: Phantom fixed to the PPS with a frame adaptor.

**FIGURE 2.**
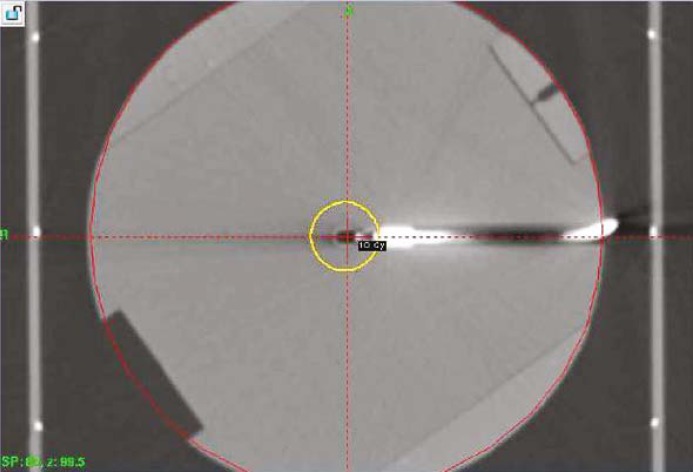
CT image of framed spherical phantom. The three fiducial marks are used to determine the treatment coordinates for the center of the spherical phantom. The phantom was imaged with an ionization chamber placed in the cylindrical cavity of the chamber cassette. The 50% isodose line for a shot with the 16 mm collimator is shown.

During the period over which measurements for this study were performed, the dose rate at the center of a spherical calibration phantom (Elekta, Atlanta, GA) for all sectors aligned with the 16 mm collimator ranged from 3.425 to 3.218 Gy/min. Rather than using the plastic connectors and red dosimetry adaptors to attach to the frame adaptor, a standard Elekta stereotactic frame (Leksell Coordinate Frame G, Elekta, Atlanta, GA) was applied to the phantom in order to more accurately simulate the conditions of a typical patient treatment. A CT fiducial box was attached to the framed phantom and subsequently imaged with a GE HiSpeed FX/i CT scanner (GE Healthcare) (Figure 1A, B). Images (1 mm slice thickness) were exported to and registered in the GammaPlan^™^ (Version 8.3.1, Elekta, Atlanta, GA) treatment planning system. The planning target was selected to be the center of the spherical phantom, where a dose of 10 Gy to the 50% isodose line was prescribed (Figure 2). For delivery of treatment plans, the frame adaptor was attached to the phantom and coupled to the PPS. Within the phantom, a calibrated 0.07 cm^3^ cylindrical ionization chamber (model PR-05P, Capintec, Ramsey, NJ) was positioned using a chamber cassette and connected to an electrometer (35617EBS Programmable Dosimeter, Keithley Instruments, Cleveland, Ohio), enabling measurements of dose during irradiation of the phantom. All measurements were taken multiple times to enable statistical analysis. The error represented in all the presented data indicates the standard deviation of the mean of these multiple, independent measurements (Type A error evaluation). The collected shutter data are presented as a dose rate (Gy/min) in the graphs and tables. Representing the shutter effect as Gy/shot (or Gy/reposition) would not be reflective of the differences between the shutter effects for plans with various shots because of its dependence on the source activity, which decays exponentially with time. As the data were acquired over an extended period of time, it seemed most appropriate to compare the shutter effect in terms of a dose rate (Gy/min or cGy/min) that is normalized to the focus dose rate (with the 16 mm collimator). For the shutter dose at the target site, we also present the effect in Gy/shot to enable users of the Perfexion^™^ to compute the expected shutter dose for their unit by multiplying shutter dose per reposition values with the ratio of dose rates (dose rate on current day to dose rate on the day the original experiment was performed).

### Measuring shutter dose to the target

Measurements of shutter doses were carried out for single run treatment plans developed to deliver the same dose to the isocenter with varying numbers of shots (1, 5, 20, 30, and 50) for the 8 and 16 mm collimators. Each run was timed with a stopwatch; the mean time difference between the single shot run and each multiple shot run was used to determine the shutter dose rate. Statistical analysis for the time measurements with the stopwatch were performed with Type A error analysis; the standard deviations of the mean times were calculated to indicate error in this study. The differences between the dose measured for the single shot run and the multiple shot runs for the same prescription dose represent the additional doses to the target site from radiation sector motions. This experimental design eliminates the transportation dose (defined above) because all plans require the same transport from the setup position to the “beam on” position and back. Therefore, the transport dose cancels when the shutter dose is calculated. The shutter effect for the 4 mm collimator was not measured at the target due to concerns with the partial volume effect associated with ionization chamber irradiation.

### Measuring shutter dose to the periphery

The shutter effect at the periphery was measured to determine dose to normal tissue, or non-target sites, within the cranium. These measurements were taken for the 4, 8 and 16 mm collimators with the ion chamber. For this study, the spherical phantom had to be framed twice with different orientations to enable measurement of radiation dose along the x-axis (lateral) and the z-axis (cranial-caudal). As a result of the symmetric shape of the collimator assembly, additional dose along the y-axis (anterior-posterior) is expected to be the same as the dose along the x-axis. To measure the dose along the x-axis, a customized cassette with the insert for the ionization chamber was aligned in the transverse plane ensuring the alignment of the cylindrical cavity for the ionization chamber along the x-axis. Though use of the plastic connectors and red dosimetry adaptor allow orientation of the cylindrical cavity along the x- and z-axes for the Perfexion^™^, this is not possible with the similar calibration setup with the model 4C. Because we compare measurements of these two units, constancy between the methods of measurement is essential for proper evaluations. In addition, the red dosimetry adaptor has been shown to cause unintended attenuation of about 1.0%.^16^ Any attenuation resulting from the frame posts will be the same for all measurements.

Measurements were acquired with the ionization chamber center positioned at 0, ±1.2, ±3.2 and ±6.2 cm from the target along the x-axis. Doses for 1, 5, 20 and 50 shot runs were measured at these points along the x-axis to determine the off axis shutter dose. Measurements along the z-axis required the cylindrical cavity to be aligned along the couch longitudinal axis. Along the z-axis, doses for 1, 5, 20 and 50 shot runs were obtained with the ionization chamber centered at 0, ±1.2, ±3.2 and ±6.2 cm from the target to determine the shutter effects to each position. Cylindrical Lucite rods (1, 2 and 3 cm long) of 6 mm diameter were used to position the chamber off-axis. The rods were inserted followed by the chamber, displacing the chamber from the phantom center by the length of the rod(s). With these rods, the chamber can be positioned at 1.2, 3.2 and 6.2 cm from the center of the phantom. The additional 0.2 cm comes from the incomplete insertion of the cylindrical rods at the end of the cavity (in the center of the spherical phantom).

## Results

### Shutter dose to the target

**FIGURE 3.**
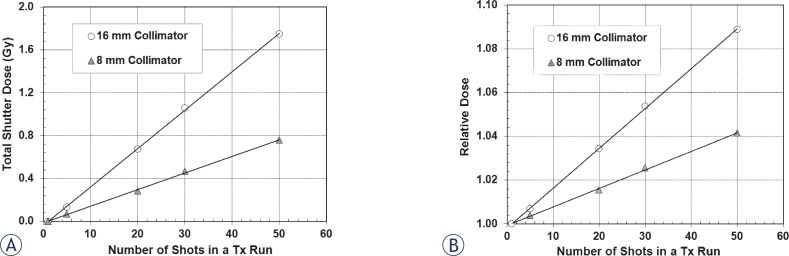
Shutter dose for the 8 and 16 mm collimators. A: The target position is the same for all measurements. Measured dose associated with PPS positioning increases with increase in the number of shots in a run and collimator size. B: The relative dose was calculated by normalizing the dose from the multiple shot plans to the single shot plan.

Figure 3A shows that shutter dose increases with frequency of repositioning and with collimator size. Dose increases of 1.59 ± 0.04 cGy and 3.53 ± 0.04 cGy per reposition were observed for the 8 and 16 mm collimators, respectively. In the extreme case of a 50 shot plan, this represents 75.6 and 174.7 cGy extra dose to the target, compared to the entire planned dose being delivered in a single shot. The shutter dose rate for each of the collimators is listed in Table 1 along with its value relative to the focus dose rate for the day of measurement (3.425 Gy/ min for the 16 mm collimator). Figure 3B shows the relative shutter dose for the 8 and 16 mm collimators.

### Shutter dose to the periphery

**FIGURE 4.**
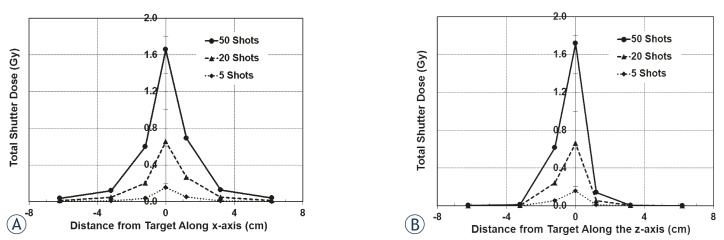
Total shutter dose for the 16 mm collimator. A: The shutter dose along the x-axis is greatest at the target site and falls off with distance; it will also increase with the number of shots. B: The shutter dose along the z-axis does not have the same symmetry as it does along the x-axis. The collimators are angled towards the inferior direction; this will increase exposure inferior to the target, as seen.

Plotted in Figure 4A is the shutter dose along the x-axis for the 16 mm collimator measured using an ion chamber. There are three separate data sets; each represents a different number of shots in a single run. The greatest shutter dose is at the target site, and falls off nearly symmetrically with increasing distance from the target site. Shutter dose to the periphery increases with increasing number of shots in a run, as is the case with shutter dose to the target site. The average shutter dose per reposition at the target is 3.70 ± 0.04 cGy and falls off symmetrically with distance along the x-axis. Table 2 gives the transit dose rates along the x-axis. Also listed are the percent dose rates (shutter dose rate as a function of the dose rate at the focus point on the day of measurement, which is 3.351 Gy/min for the 16 mm collimator).

**FIGURE 5.**
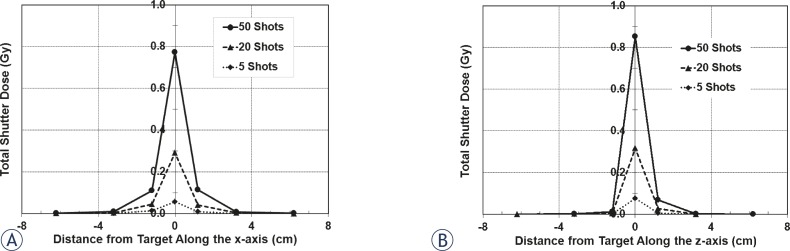
Total shutter dose for the 8 mm collimator. A: Measurements along the x-axis. B: Measurements along the z-axis.

Along the z-axis, there is not the same symmetry in shutter dose as seen with dose measurements along the x-axis (Figure 4B). The dose is still highest in magnitude at the target site and falls-off on each side; however, the falloff is steeper in the superior region from the target. The shutter dose per reposition is 3.78 ± 0.10 cGy for the target, while at 6.2 cm on each side of the target, it is nearly zero. Table 2 lists the shutter dose rates along the z-axis; also presented is the percent of the focus dose rate on the day of measurement (3.366 to 3.362 Gy/min with the 16 mm collimator). These measurements were also taken for the 8 mm collimator to determine the shutter effects from the radiation sector motions to these collimators. Figure 5 shows the total shutter dose for the x- and z-axes and Table 3 shows the dose rates for each position measured.

**FIGURE 6.**
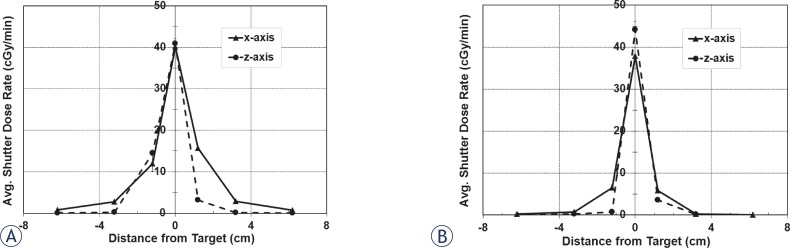
A comparison of the average shutter dose rates along the x- and z-axes for the A: 16 mm and B: 8 mm collimators. The difference in dose rates at target site may be due to the orientation of the ionization chamber for each measurement.

The average shutter dose rates were calculated and plotted for positions along both axes for the 16 mm collimator (Figure 6A). The difference in dose profiles in each axis can be attributed to the inferiorly focused orientation of the beamlets. The discrepancy in transit doses at the target site is due to the non-isotropic isodose distribution at isocenter, where the dose is weighted more in the superior direction. When the ionization chamber is placed at the target, the exposure integrated over the collecting volume will be greater with the ionization chamber oriented in the z-direction (cranial-caudal) than if oriented along the x-direction (left-right). The average shutter dose rates were also calculated and plotted for positions along both axes for the 8 mm collimator (Figure 6B).

## Discussion

### Measured data

The shutter dose to the target increases with increasing number of shots and collimator size, as shown in Figure 3A. The measured doses for the multiple shot plans were normalized to the expected dose (measured for the single shot plan) and graphed in Figure 3B. There are several factors that contribute to the observed deviation in relative shutter effect for the 16 and 8 mm collimator. The shutter time is longer for the 16 mm collimator (5.38 seconds) than the 8 mm collimator (2.39 seconds), and the collimator size is considerably larger. This means once the sources begin to align with the open collimators, it takes longer to reach alignment, thus depositing more unaccounted dose. These factors contribute a greater measured shutter effect for the 16 mm collimator. Another source of contribution to the shutter dose for the 16 mm collimator is an additional source of radiation. As the sources move from the “sector off” position to the 16 mm collimator position, the sources must flash over the open 4 mm collimator; when the sources retract after the shot is complete, there is another flashing occurrence over the 4 mm collimator. Thus the effective shutter dose from use of the 16 mm collimator comes from both the 4 mm and 16 mm collimators. This passing motion would contribute additional dose, resulting in a larger relative shutter effect from the 16 mm collimator measurements than expected.

Multiple shots in a run will result in additional, unaccounted exposure of surrounding normal tissue from the shutter effect because the GammaPlan^™^ software does not fully account for the sector motions accompanying PPS repositioning. This is especially true for plans that require larger collimator sizes and greater number of shots. The amount of peripheral exposure during repositioning will depend on location – proximity to the target site means greater exposure from the shutter effect.

There are other considerations that may change the total unaccounted dose during treatment. The PPS does not change coordinates until the sectors reach the “sector off” position; this minimizes the exposure from leakage and scatter dose. Because our experimental design does not include actual position change through PPS motions, there may be added dose from leakage and scatter when there are changes in treatment coordinates during an actual treatment. The activity of the sources affects the leakage and scatter radiation to the patient during coordinate repositioning; higher activity sources will contribute more unintended exposure to the patient during positioning than lower activity sources.

The shutter dose rate is dependent on the activity of the sources in a predictable manner over time. It may also depend on the time it takes for the sectors to move from the open collimator position to the off position; this value may not be the same for all Perfexion^™^ units. In an actual patient treatment, the unaccounted dose will also depend upon the time needed for the PPS to transit between treatment coordinates. With the hybrid shot capability of the Perfexion^™^, shutter dose may depend on the combination of collimation that makes up a shot.^17^ Finally, the maximum number of shots used in this study is 50 in a single treatment run. If more shots and runs are required, the unaccounted dose may be more than reported here.

For these measurements, the target site for all shots is fixed to the unit isocenter at (100, 100, 100). However, a typical treatment plan will have shots that are distributed to cover a volume that encompasses the target site, where none of the shots are overlapping. The excess target and peripheral dose rates for each collimator can be applied to each shot of a treatment plan to determine the overall distribution of excess dose from shutter with the Perfexion^™^. The shape of a treatment target can vary extensively; in addition, treatments will also depend on the bias of the planner. Because there are no standard treatment plans for an irregularly shaped target and because of the subjectivity with planning, formulating a treatment plan for a hypothetical target volume gives no indication on the effect of shutter to other treatment plans. Rather, the additional target and peripheral dose from shutter per shot for each collimator can be used and applied to the position of each shot to determine the distribution of additional dose from shutter. That is, using the coordinates of each shot, the shutter dose profile can be applied to their respective shot to map the shutter dose distribution for a treatment volume. Figures 4, 5 and 6 show the shutter dose profiles and display the shutter effects to regions peripheral to the target site.

### Perfexion^™^ *vs.* model 4C

Gamma Knife radiosurgery is a highly precise stereotactic tool for the treatment of intracranial disease.^18^–^22^ The introduction of the Automatic Positioning System (APS) with the model C appreciably streamlined the dose delivery process by enabling delivery of multiple shots within a single treatment run.^19^,^20^,^23^ The APS is an analogous device to the PPS; it repositions the patient’s head to allow therapeutic dose delivery to target site(s), though with a much smaller coordinate repositioning range than the PPS.^1^–^3^ Repositioning with the APS also has an element that contributes unaccounted exposure – the intershot transit effect.^13^ A similar study was conducted with the APS of the Gamma Knife^®^ model 4C using an ionization chamber as the dosimeter.^15^ As a part of this work, we compare the shutter effect of the Perfexion^™^ with the inter-shot transit effect of the model 4C previously measured.

**FIGURE 7.**
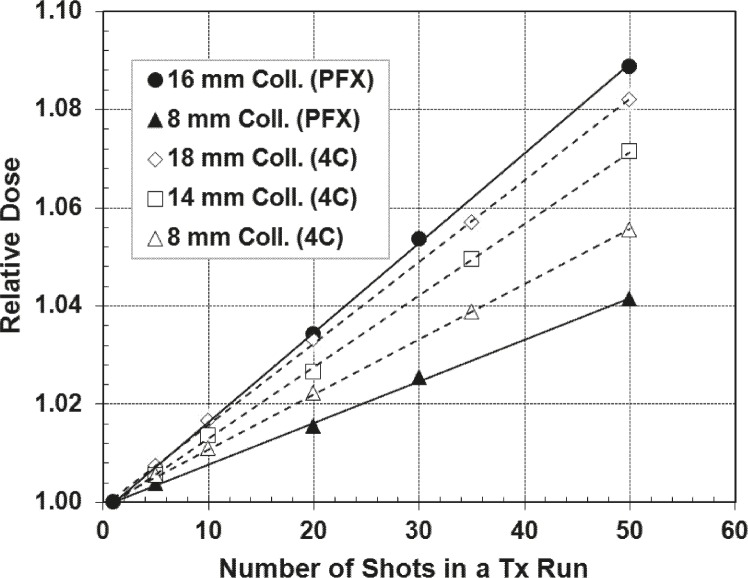
Shutter and inter-shot transit dose at the target site for the Gamma Knife^®^ Perfexion^™^ (PFX) and model 4C (4C), respectively. The shutter and inter-shot transit doses are not accounted for by the treatment planning system.

With the model 4C, inter-shot transit dose rates were measured for the 8, 14 and 18 mm collimators. To compare the data from the model 4C with the Perfexion^™^, the shutter and transit dose rates were normalized using the calibrated focus dose rates for the day of measurement. Table 1 shows the transit and shutter dose rates relative to the focus dose rates. For all collimator sizes, the Perfexion^™^ has a greater shutter dose rate than the transit dose rate of the model 4C; however, the shutter doses are comparable to the transit doses. The differences between the dose rates can be attributed to the shorter time for the radiation sectors to move from a collimator to the “sector off” position (for the Perfexion^™^) than the time for the couch to move from the focus to defocus position (of the model 4C). Though the design of each model is different, the shutter doses are comparable to the intershot transit doses. Figure 7 shows the relative shutter and transit doses to the target for each model; the added dose is collimator and shot dependent. Comparing the additional dose measured for the 8 mm collimator for both models, the doses per reposition are nearly identical (Table 1). Of course, if the calibrated focus dose rate of the model 4C were the same as the Perfexion^™^ (2.254 versus 3.425 Gy/min, respectively) then the added dose would probably be larger for the model 4C. The activity of the sources will affect the unaccounted dose.

In Table 2, the shutter dose rates for the 16 mm collimator of the Perfexion^™^ and the transit dose rates for 18 mm collimator helmet of the model 4C are compared along the x- and z-axes. As a fraction of the focus dose rate, the shutter effect for the Perfexion^™^ is larger than the transit effect of the model 4C along the x-axis. However, along the z-axis, the corresponding effect is more substantial for the model 4C in regions superior to the target than with the Perfexion^™^. This can be attributed to the helmet and repositioning design of the model 4C. Greater transit dose rates are seen at more superior regions within the phantom because of proximity to the sources. Also, as the helmet moves away from and towards the sources during repositioning, the unfocused beam will intersect the fiberglass helmet cap (where there is little attenuation of the beam) exposing the superior region of the phantom or patient to unintended radiation.^24^–^25^ The transit dose increases at positions closer to the crown of the head because of the poorly shielded 23 cm diameter opening at the helmet’s apex, which results in more exposure from leakage and scatter to the superior regions of the phantom.^15^,^24^ This is the reason for the behavior of the increased transit dose towards the superior portion of the z-axis for the model 4C. With the Perfexion^™^, this is not observed because the radiation sectors are the components of the unit that move in order to reduce exposure during repositioning, not the couch.

**FIGURE 8.**
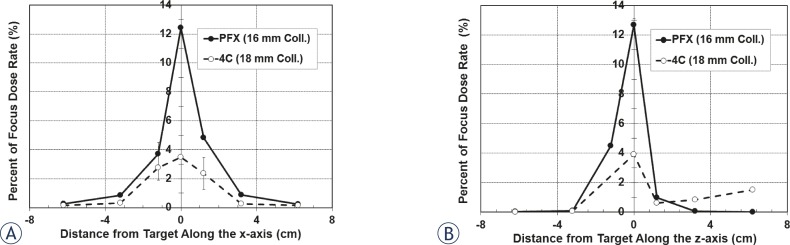
A comparison of the shutter dose rate (as a percent of the focus dose rate with the 16 mm collimator) for the Perfexion^™^ to the inter-shot transit dose rate (as a percent of the focus dose rate with the 18 mm collimator) for the model 4C. A: Along the x-axis, the shutter and transit dose rates are similar in behavior, but the shutter effect with the Perfexion^™^ is greater than the transit effect with the model 4C. B: The effect is different between the two models in the superior region, along the z-axis. The dose falls off from the target position then increases with the model 4C; the falloff of the shutter dose is sharper in the superior region for the Perfexion^™^. This can be attributed to the difference in machine design and treatment coordinate change between the two models.

Figure 8A and 8B plot the behavior of the shutter and transit effect as a function of the calibrated focus dose rate along both x- and z-axes for their respective model. Figure 8A shows a similar trend between each model, with the major difference seen with magnitude. In Figure 8B, a difference in the region superior to the target can be seen, which can be attributed to the difference in design of each model. The model 4C has a poorly shielding helmet cap that allows contribution of additional dose. In terms of limiting the shutter dose to the target, there is an improvement with the latest model. Additional dose to the target site is not a vital issue because when planning a treatment, the limitation is the dose to the peripheral structure, especially critical structures. The focus on shutter dose is therefore not because of significant concern of added dose to the target, but rather, additional dose to the periphery of the target. Accounting for this shutter effect would better document dose to peripheral structures as well as improve dosimetric accuracy of the treatment plan.

### Previous studies

A study of the relative output factors of the 4 and 8 mm collimators of the Perfexion^™^ was reported by Novotny *et al*.^9^ To correct for the relative output factors, the authors also report the transition doses (which we define as the shutter dose) for all three collimators in this study: 0.98, 1.51, and 3.46 cGy for the 4, 8 and 16 mm collimators, respectively.^9^ This was measured with an ionization chamber in the spherical phantom using the red dosimetry adaptor. These values are consistent with our values for shutter measured with the 8 and 16 mm collimators (1.59 and 3.53 cGy per reposition, respectively, as seen in Table 1); however, no dose rate for the original experiment is reported in their article so we are unable to conclusively compare our data.

In the Perfexion^™^ manual, a value is given for the shutter dose for the 4 mm collimator; however, there are no indications of the method used to obtain this value. The magnitude of the shutter effect for a dose rate of 3.0 Gy/min is 0.005 Gy per reposition (or 0.5 cGy/reposition). This value is approximately half the value of that reported by Novotny *et al*.

Ruschin *et al*. conducted a thorough investigation of peripheral dose from the treatment of large lesions with the Perfexion^™^.^11^ They conducted measurements studying the effect of the target’s volume and collimator size on peripheral exposures.^11^ Many of these plans were generated with a significant number of shots to adequately cover the target site with the appropriate dose prescription, but contribution from the shutter effect is not considered in their study. Given the positions of each shot, the values we measured for peripheral shutter dose can be used to determine the overall shutter dose distribution and contribution to their measured data.

## Conclusions

For multiple shot runs, radiation sector motions result in additional dose to the target site and its periphery due to the shutter effect. The relationship between unaccounted dose and collimator size, shutter dose and number of repositions, and the positional dependence of the shutter dose to the focus are reported. The shutter dose rates are greater with the Perfexion^™^ than with the model 4C, but the shutter doses are comparable to the intershot transit dose. Though regarded as a highly accurate modality for intracranial radiosurgery, there is still potential for substantial unaccounted dose during treatment resulting from radiation sector motions accompanying PPS repositioning. This may be important for treatment areas around critical structures within the brain. Further characterization of exposure from the radiation sector motions accompanying movement of the PPS and better documentation of these radiation doses would improve the accuracy of the calculated treatment plans.

## Figures and Tables

**TABLE 1.** Shutter and inter-shot transit dose rates at the target. The shutter dose rate is represented as a percent of the focus dose rate on the day of measurements (Perfexion^™^ had a focus dose rate of 3.425 Gy/min for the 16 mm collimator; model 4C had a focus dose rate of 2.254 Gy/min for 18 mm collimator helmet).

**Perfexion^™^: Collimator Size and Shutter Dose Rates**			

**Collimator Size (mm)**	**Shutter Dose Rate (cGy/min)**	**Percent of Focus Dose Rate (%)**	**Shutter Dose per Reposition (cGy)**

16	40.04 ± 0.51	11.69 ± 0.15	3.53 ± 0.04
8	39.52 ± 1.08	11.54 ± 0.31	1.59 ± 0.04

**Model 4C: Collimator Size and Inter-shot Transit Dose Rates**			

**Collimator Size (mm)**	**Transit Dose Rate (cGy/min)**	**Percent of Focus Dose Rate (%)**	**Transit Dose per Reposition (cGy)**

18	8.81 ± 0.41	3.91 ± 0.18	2.37 ± 0.00
14	6.98 ± 0.51	3.10 ± 0.23	1.94 ± 0.00
8	5.89 ± 0.51	2.62 ± 0.23	1.58 ± 0.00

**TABLE 2.** Shutter dose rates (Perfexion^™^) and Inter-shot Transit dose rates (model 4C) along the x and z-axes. The shutter dose rate is also represented as a percentage of the focus dose rate on the day of measurement (Perfexion^™^ had focus dose rates ranging from 3.366 to 3.351 Gy/min for the 16 mm collimator; model 4C had a focus dose rate of 2.220 Gy/min for 18 mm collimator helmet).

**Dose Rates Along the x-axis**				

	**Perfexion^™^ (16 mm Collimator)**		**Model 4C (18 mm Collimator)**	

**Position (cm)**	**Shutter Dose Rate (cGy/min)**	**Percent of Focus Dose Rate (%)**	**Transit Dose Rate (cGy/min)**	**Percent of Focus Dose Rate (%)**

−6.2	0.91 ± 0.08	0.27 ± 0.02	0.36 ± 0.06	0.16 ± 0.03
−3.2	2.88 ± 0.08	0.85 ± 0.02	0.70 ± 0.04	0.32 ± 0.02
−1.2	12.51 ± 2.63	3.72 ± 0.62	6.11 ± 1.85	2.75 ± 0.83
0	41.80 ± 0.55	12.42 ± 0.12	7.72 ± 0.08	3.48 ± 0.04
1.2	16.30 ± 0.07	4.84 ± 0.02	5.28 ± 2.45	2.38 ± 1.10
3.2	3.03 ± 0.08	0.90 ± 0.02	0.59 ± 0.02	0.27 ± 0.01
6.2	0.79 ± 0.12	0.23 ± 0.02	0.36 ± 0.04	0.16 ± 0.02

**Dose Rates Along the z-axis**				

	**Perfexion^™^ (16 mm Collimator)**		**Model 4C (18mm Collimator)**	

**Position (cm)**	**Shutter Dose Rate (cGy/min)**	**Percent of Focus Dose Rate (%)**	**Transit Dose Rate (cGy/min)**	**Percent of Focus Dose Rate (%)**

−6.2	0.05 ± 0.03	0.02 ± 0.01	0.00 ± 0.00	0.00 ± 0.00
−3.2	0.28 ± 0.04	0.08 ± 0.01	0.10 ± 0.10	0.05 ± 0.05
−1.2	15.09 ± 0.28	4.48 ± 0.08	Not Measured	Not Measured
0	42.63 ± 1.12	12.66 ± 0.33	8.66 ± 0.19	3.90 ± 0.08
1.2	3.27 ± 0.21	0.97 ± 0.06	1.37 ± 0.30	0.62 ± 0.14
3.2	0.15 ± 0.03	0.04 ± 0.01	1.88 ± 0.14	0.85 ± 0.06
6.2	0.03 ± 0.02	0.01 ± 0.00	3.34 ± 0.04	1.51 ± 0.02

**TABLE 3.** Shutter Dose Rates along the x-axis for the 4 and 8 mm collimators. For these measurements, the focus dose rate ranged from 3.230 to 3.221 Gy/min. The shutter dose rate is represented as a percent of the focus dose rate on the day of measurement for the 16 mm collimator.

**Dose Rates Along the x-axis**				

	**4 mm Collimator**		**8 mm Collimator**	

**Position (cm)**	**Shutter Dose Rate (cGy/min)**	**Percent of Focus Dose Rate (%)**	**Shutter Dose Rate (cGy/min)**	**Percent of Focus Dose Rate (%)**

−6.2	0.00 ± 0.09	0.00 ± 0.01	0.22 ± 0.22	0.07 ± 0.09
−3.2	0.65 ± 0.04	0.20 ± 0.02	0.74 ± 0.09	0.23 ± 0.04
−1.2	4.96 ± 0.16	1.53 ± 0.07	6.50 ± 0.65	2.01 ± 0.25
0	Not Measured	Not Measured	37.86 ± 0.28	11.72 ± 0.14
1.2	1.74 ± 0.25	0.54 ± 0.09	5.81 ± 0.27	1.80 ± 0.11
3.2	0.35 ± 0.13	0.11 ± 0.05	0.18 ± 0.29	0.06 ± 0.07
6.2	−0.23 ± 0.15	−0.07 ± 0.05	0.03 ± 0.07	0.01 ± 0.02

**Dose Rates Along the z-axis**				

	**4 mm Collimator**		**8 mm Collimator**	
**Position (cm)**	**Shutter Dose Rate (cGy/min)**	**Percent of Focus Dose Rate (%)**	**Shutter Dose Rate (cGy/min)**	**Percent of Focus Dose Rate (%)**

−6.2	−0.08 ± 0.17	−0.03 ± 0.05	−0.03 ± 0.58	−0.01 ± 0.15
−3.2	0.03 ± 0.12	0.01 ± 0.02	0.24 ± 0.57	0.07 ± 0.23
−1.2	2.15 ± 0.11	0.66 ± 0.05	0.66 ± 0.57	0.21 ± 0.23
0	Not Measured	Not Measured	44.16 ± 1.84	13.67 ± 0.83
1.2	0.12 ± 0.01	0.04 ± 0.01	3.57 ± 0.57	1.10 ± 0.23
3.2	0.06 ± 0.22	0.02 ± 0.05	0.08 ± 0.57	0.02 ± 0.22
6.2	0.16 ± 0.22	0.05 ± 0.06	−0.05 ± 0.57	−0.01 ± 0.19
